# Penetrating Neck Injury With Common Carotid Artery Involvement: A Case Report

**DOI:** 10.7759/cureus.103127

**Published:** 2026-02-06

**Authors:** Doaa A Alfraidy, Abdullah H Alhojaili, Lama H Bedaiwi

**Affiliations:** 1 Trauma and Acute Care Surgery, King Fahad General Hospital, Medina, SAU; 2 General Surgery, King Fahad General Hospital, Medina, SAU

**Keywords:** carotid artery, dual antiplatelet therapy, penetrating neck injuries, surgical intervention, trauma management

## Abstract

Penetrating neck injuries often involve vascular injuries, including the carotid arteries, posing significant morbidity and mortality risks. This report presents a case of a 23-year-old male who sustained a penetrating neck injury from a nail gun, specifically affecting the common carotid artery (CCA). Upon arrival at the emergency department, the patient exhibited hemodynamic stability, with no active bleeding or neurological deficits. Initial imaging, including CT angiography (CTA) of the neck, revealed a metallic foreign body near the CCA and air foci suggestive of a muscle tear. Following resuscitation per Advanced Trauma Life Support (ATLS) protocols, the patient underwent emergency neck exploration. Surgical findings included a small hematoma and an intimal injury to the CCA, which was repaired using sutures after nail extraction. Postoperatively, the patient showed no complications and was discharged in good condition, receiving dual antiplatelet therapy to mitigate thrombotic risks.

This case highlights the critical importance of timely assessment and intervention in managing neck vascular injuries, particularly those involving the carotid artery. Adhering to ATLS guidelines, using imaging for accurate diagnosis, and employing appropriate surgical techniques can significantly improve patient outcomes. Early recognition and management remain vital to enhancing prognosis and reducing mortality associated with these complex injuries.

## Introduction

Penetrating neck injuries account for approximately 5-10% of all traumatic injuries presenting to trauma centers. The carotid arteries are involved in approximately 5-15% of penetrating neck injuries [1].

Overall mortality of penetrating neck injuries in adults is 2-10%, and it increases significantly with major vascular injury and neurologic deficit [1]. Neck vascular injuries are challenging for surgeons due to the higher risk of morbidity and mortality [2].

Treatment strategies depend on the hemodynamic stability of the patient and the presence of hard or soft signs of vascular injury. In unstable patients or those with hard signs of vascular injury, emergent surgical intervention is indicated. In patients with mild signs of vascular injury, and in those with severe signs who are hemodynamically stable or transient responders to resuscitation, diagnostic imaging is indicated and allows identification of the vascular injury’s location, extension, and severity. Operative and nonoperative treatment is tailored for each patient according to the imaging findings [3].

Our report describes a patient who presented with a penetrating neck injury involving the common carotid artery (CCA) and the management strategy employed.

## Case presentation

A 23-year-old male, with no known chronic disease, was brought by ambulance to the emergency department after sustaining a nail injury to the neck while using a nail gun. There was no history of profuse bleeding, loss of consciousness, or breathing difficulties.

**Figure 1:**
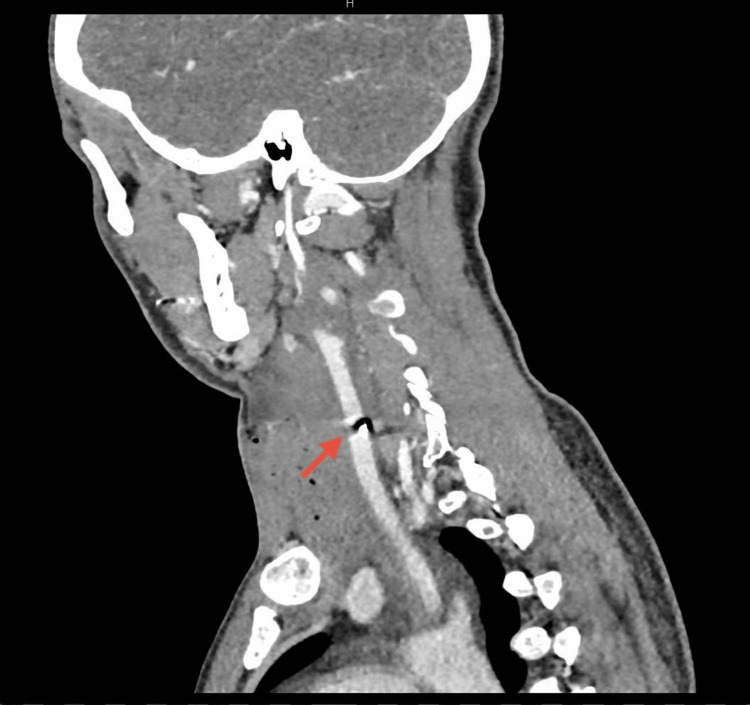
Preoperative CT. Left neck swelling showing air foci extending from the upper chest to the insertion of the left sternomastoid muscle, measuring about 7 x 10 cm, and associated with shifting of the trachea to the right, suspicious for a muscle tear. A left metallic foreign body with metallic artefact was noted very close to and abutting the common carotid artery.

**Figure 2:**
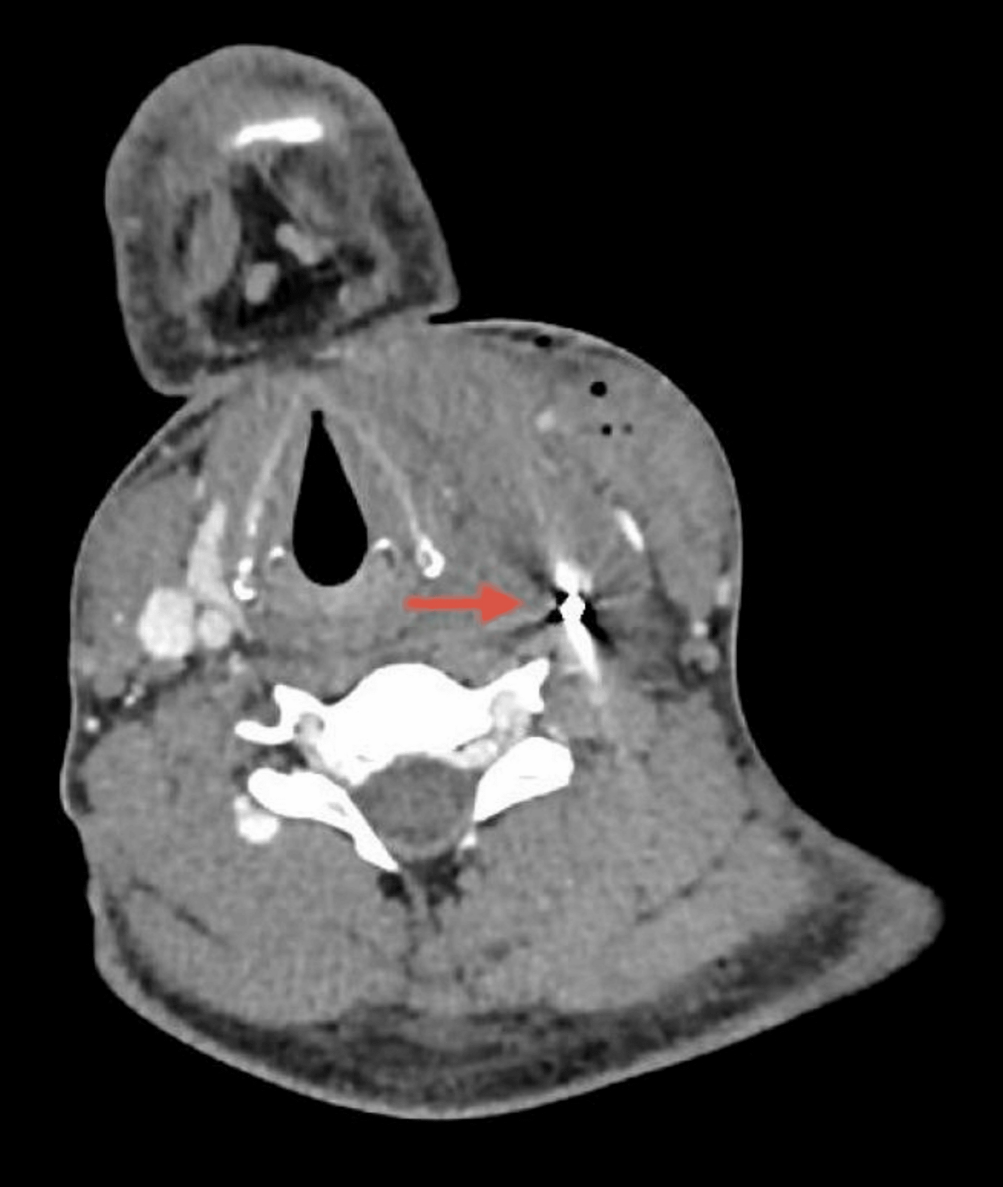
Preoperative CT. Left neck swelling showing air foci associated with shifting of the trachea to the right, suspicious for a muscle tear. A left metallic foreign body with metallic artefact was noted very close to and abutting the common carotid artery.

**Figure 3:**
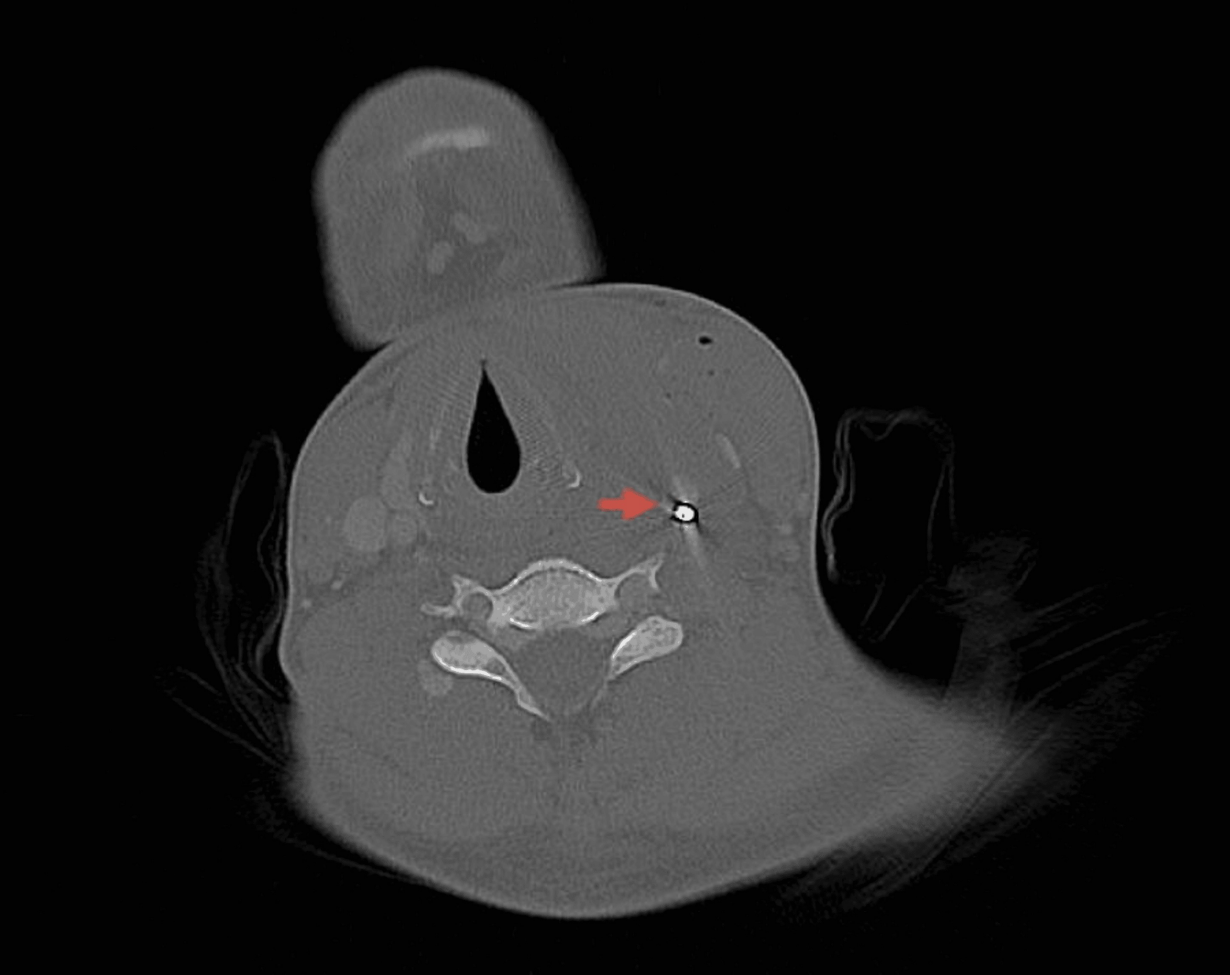
Preoperative CT. Left metallic foreign body with metallic artifact very close and abutting to the common carotid artery.

On examination, the patient arrived conscious, alert, and oriented, communicating freely and breathing spontaneously, with equal bilateral air entry. There was no active external bleeding. Vital signs were as follows: heart rate (HR) 111, blood pressure (BP) 127/98, and peripheral oxygen saturation (SpO₂) 97% on room air (RA). Exposure revealed a small inlet site measuring less than one cm in the neck, medial to the left sternocleidomastoid (SCM) in zone II. There were no hard signs of vascular injury. Only surgical emphysema was noted, with no expanding hematoma, palpable thrill, or audible bruit. Neurological examination revealed a Glasgow Coma Scale (GCS) score of 15/15, with no signs of lateralization or focal deficit. Cranial nerve examination was intact. An extended focused assessment with sonography for trauma (E-FAST) examination was performed and was negative. Chest X-ray showed no abnormalities. Blood investigations are shown in Table 1, and a neck CT angiogram was performed as shown in Figures 1-3. 

**Table 1:** Blood test results. Other blood investigations were within normal ranges . WBC: white blood cell, HGB: haemoglobin, BUN: blood urea nitrogen.

Test	Result	Unit	Min normal range	Max normal range
WBC	7.7 x 10^9^	/L	4	10
HGB	15.1	g/dL	13	17
BUN	4.9	mmol/L	2.5	8.3
Creatinine	75	μmol/L	44	115

Management

**Figure 4:**
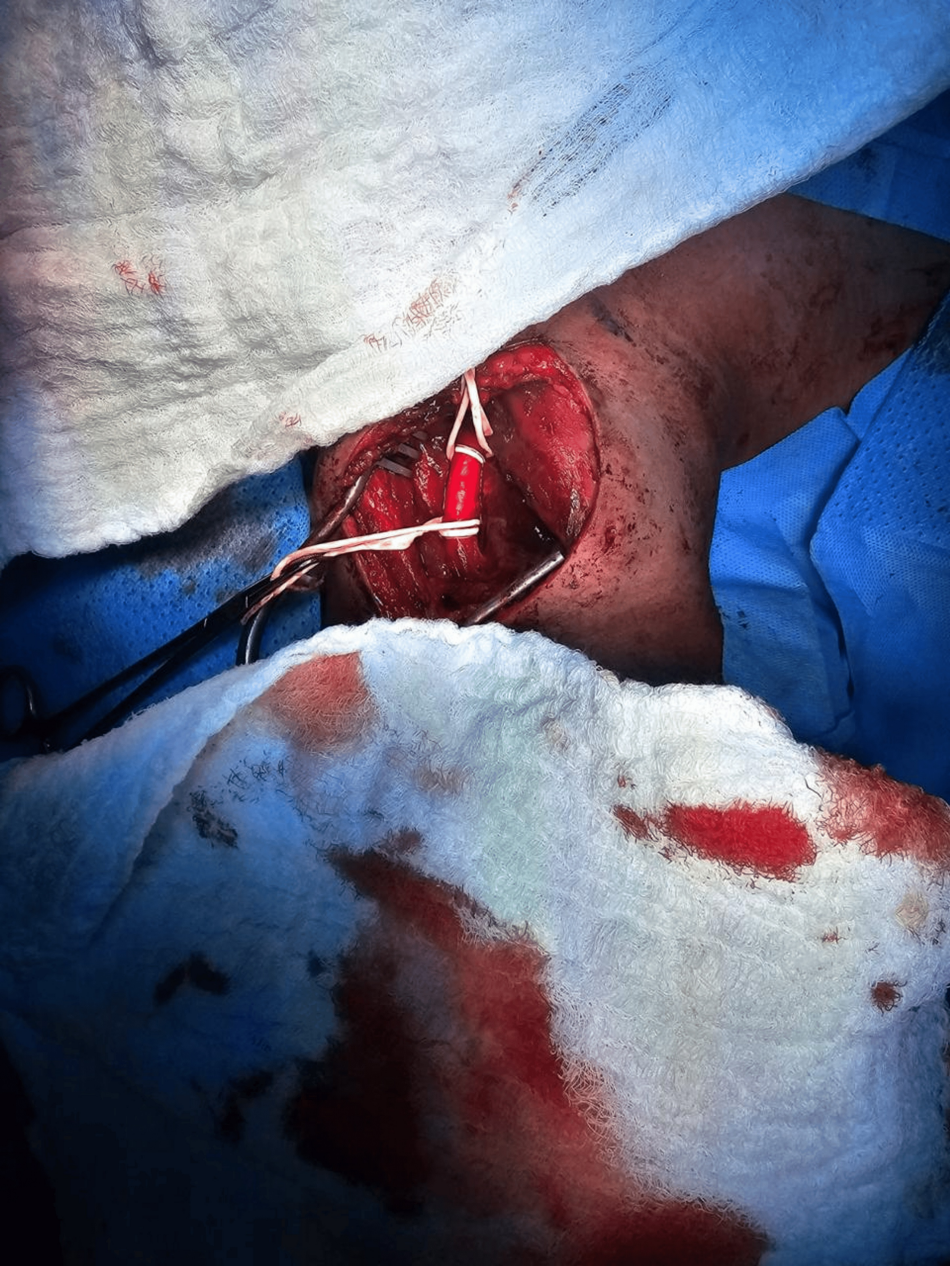
Intraoperative view prior to arteriotomy.

**Figure 5:**
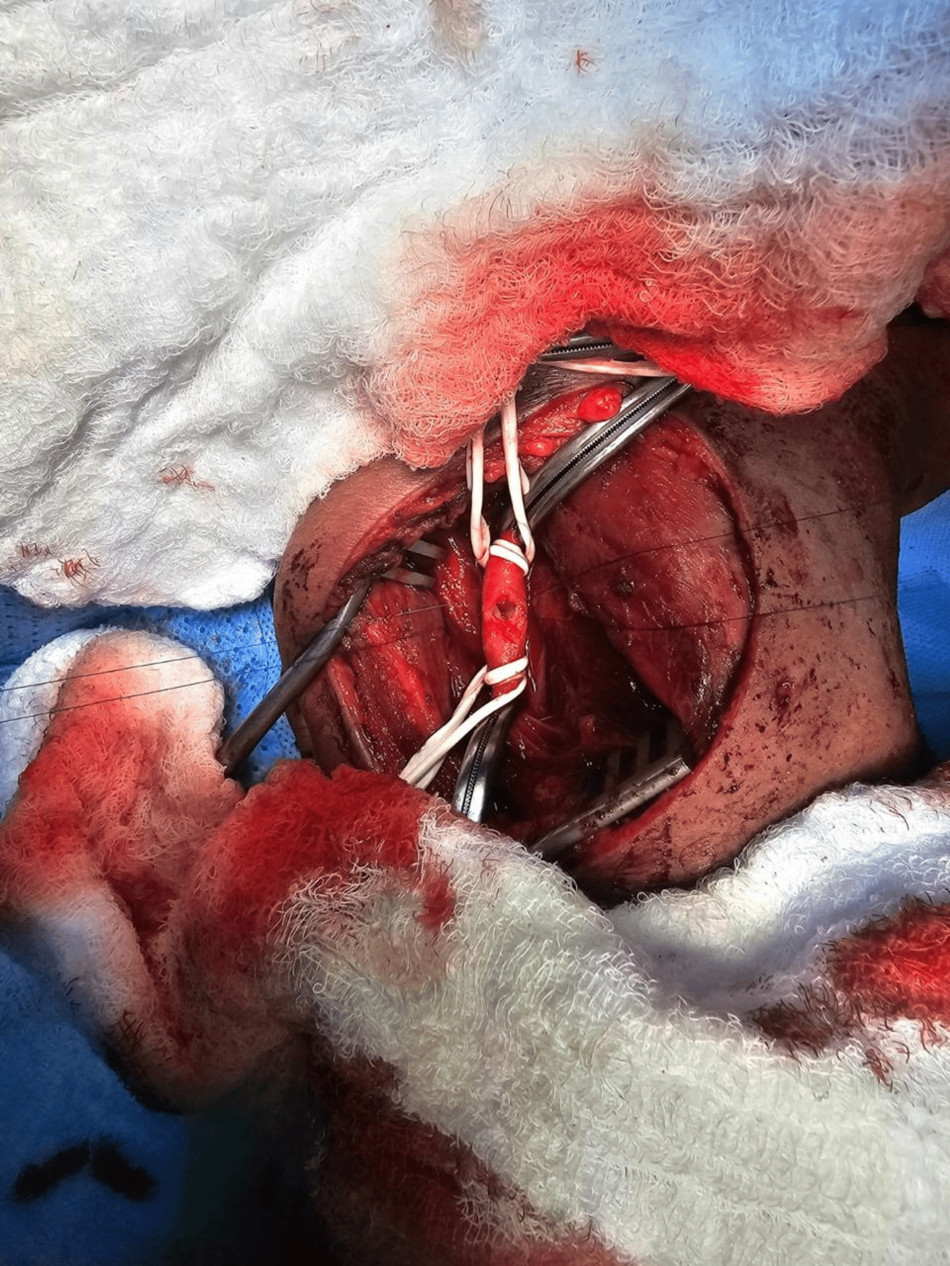
Intraoperative view (arteriotomy).

**Figure 6:**
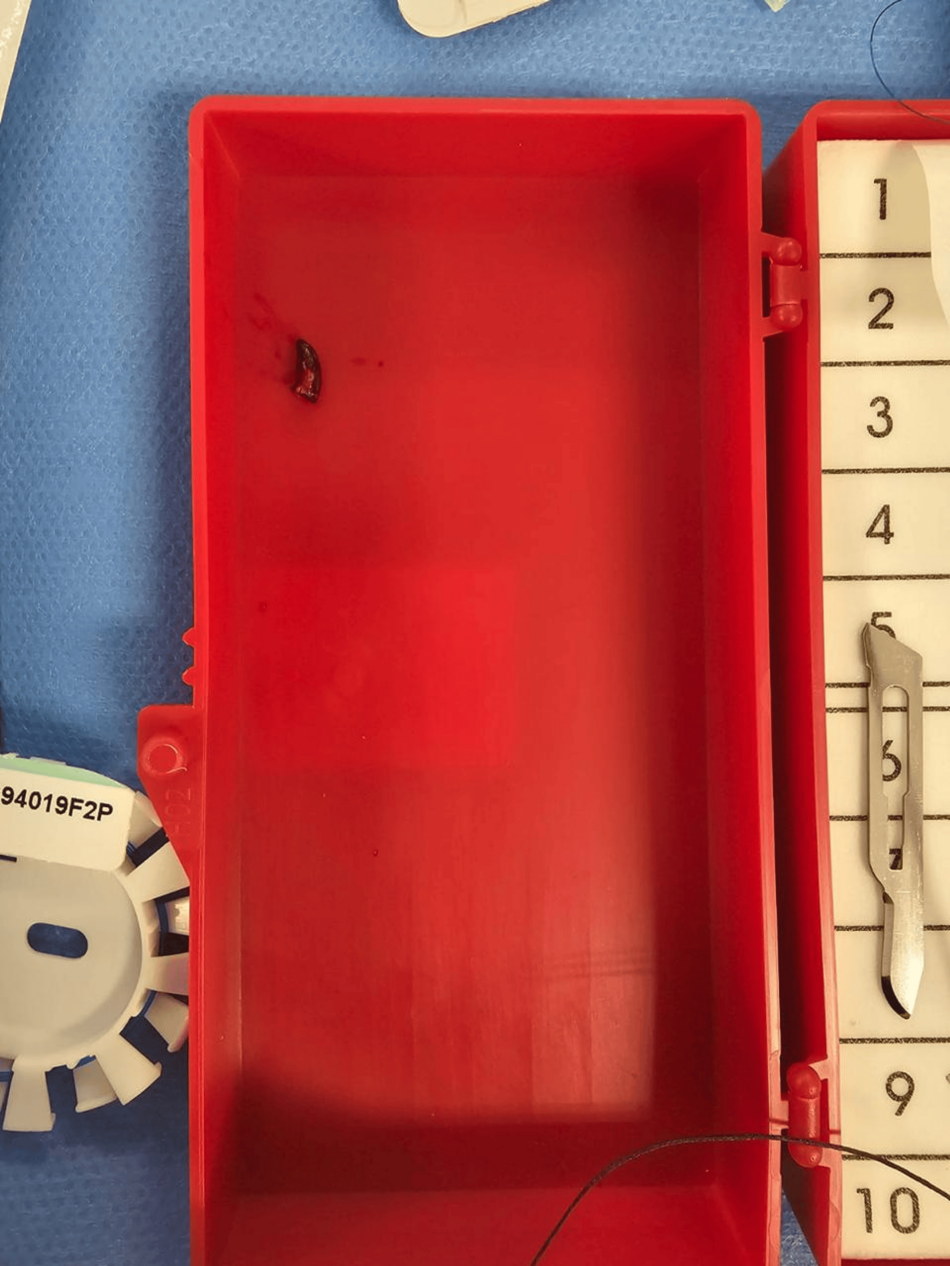
Nail after extraction.

The patient was resuscitated following the Advanced Trauma Life Support (ATLS) protocol and then taken for emergency neck exploration. An incision was made along the anterior border of the left sternocleidomastoid (SCM). The SCM was retracted laterally, and the cranial nerves and vagus nerve were identified and protected. Exploration was done for the major vessels, including the jugular vein and carotid artery, and the surrounding tissue. A small hematoma was found medial to the carotid artery, with a small injury over the anterior wall. Heparin sodium 5000 IU was administered intravenously. Vascular loops were applied proximally and distally with a vascular clamp, as shown in Figure 4. The carotid artery was opened, as shown in Figure 5, for nail extraction. The nail was inside the carotid artery and caused an intimal injury to the posterior wall but did not penetrate it. After nail extraction, as shown in Figure 6, the intimal injury was fixed using Prolene 6-0, and closure of the anterior wall injury was done using Prolene 5-0 interrupted sutures. Washing was done, hemostasis was achieved, and Surgicel was applied over the repair site. Layered closure of the platysma and skin was done, and a pressure dressing was applied. The patient was extubated and shifted to recovery in good condition. Neurological examination was intact. 

**Figure 7:**
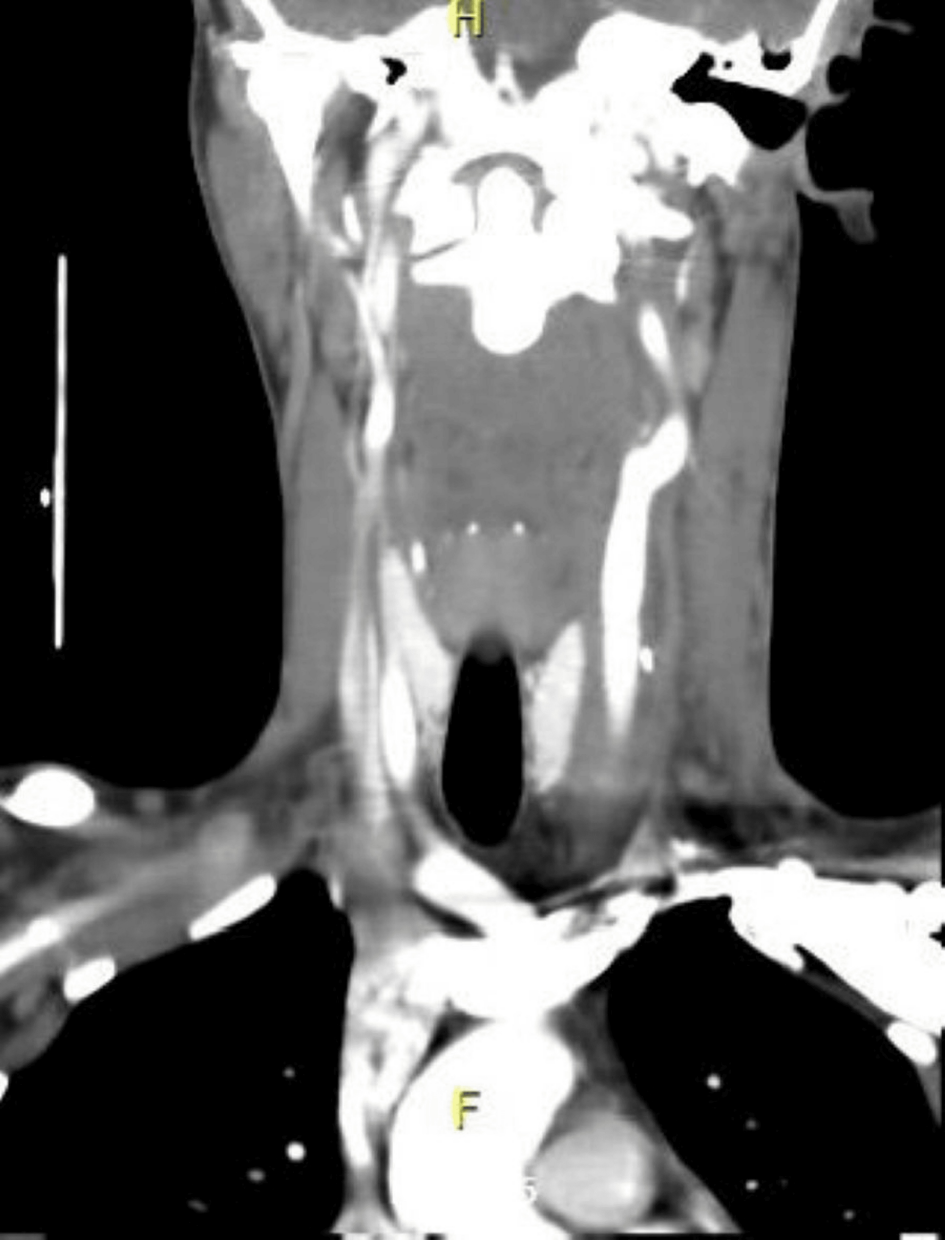
Postoperative CT (coronal plane). Postoperative changes with no obvious contrast extravasation, dissection, or wall irregularities were observed.

**Figure 8:**
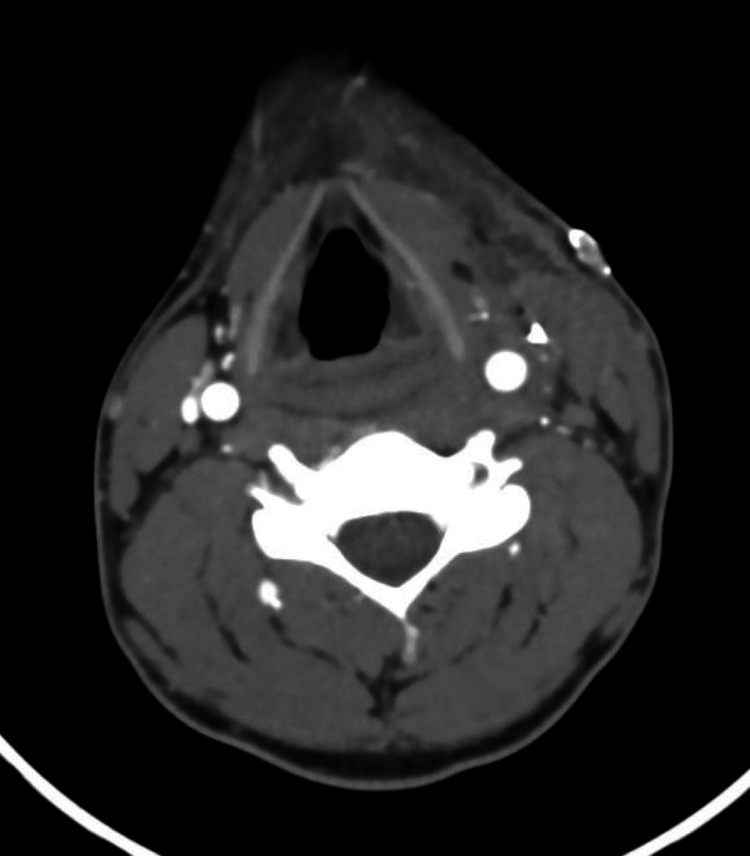
Postoperative CT (axial plane). Postoperative changes with no obvious contrast extravasation, dissection, or wall irregularities were observed.

The patient was then observed for four days in the general surgical ward. He tolerated the procedure well, and no postoperative complications were noted. He was started on dual antiplatelet therapy for three months. Neck CT angiography was performed on postoperative day four and was reported as showing postoperative changes with no obvious contrast extravasation, dissection, or wall irregularities, as shown in Figures 7, 8. 

The patient was discharged from the hospital in good condition. He was seen in the OPD two weeks following discharge and was doing well, with the wound healed well.

## Discussion

Overview of neck gunshot injuries

Neck gunshot wounds are complex injuries that can affect vital structures, including the carotid arteries, jugular veins, and cranial nerves. The management of these injuries is critical due to the potential for significant morbidity and mortality, which are summarized in Table 2 [1,4-6]. Injuries to the carotid artery can lead to life-threatening complications such as massive hemorrhage or stroke.

**Table 2:** Morbidity and mortality in penetrating neck injuries [1,4-6]. CTA: CT angiography.

Injury type	Mortality rate	Major morbidity	Key morbidity details
All neck vascular injuries (overall)	5–15%	Neurologic deficit, hemorrhage	Depends on vessel injured, hemodynamic status, and timing of repair
Carotid artery injury (overall)	10–30%	Stroke, cranial nerve injury	Stroke in 10–25% of cases
Penetrating carotid artery injury	15–30%	Ischemic stroke, pseudoaneurysm	Mortality rises to 40–60% with active bleeding or cerebral ischemia
Jugular vein injury	<5–10%	Hemorrhage, air embolism	Morbidity mainly related to massive blood loss
Vertebral artery injury	5–20%	Posterior circulation stroke	Often occult; diagnosed by CTA
Carotid sheath injury (combined)	10–20%	Cranial nerve IX–XII injury	May result in permanent neurologic deficits
Gunshot-related neck vascular injury	Higher than stab wounds	Severe neurologic injury	High-energy tissue destruction increases stroke and death
Delayed diagnosis (>24 h)	Significantly increased	Stroke, death	Stroke rates up to 30–40%

Physical examination and resuscitation

The approach to penetrating neck trauma, especially carotid artery injuries, has evolved with time. The initial evaluation and management of neck injuries should primarily emphasize physiological stabilization in line with Advanced Trauma Life Support (ATLS) protocols, as well as damage-control surgery. A meticulous physical examination is regarded as a reliable and safe method for assessing vascular injuries. The presence of hard signs on initial assessment, outlined in Table 3, indicates the need for emergent surgery [4]. 

**Table 3:** Hard and soft signs of penetrating neck injuries [4].

Hard signs	Soft signs
Active arterial bleeding	History of significant hemorrhage at scene
Expanding or pulsatile neck hematoma	Stable, non-pulsatile hematoma
Bruit or thrill over the wound	Neurologic deficit not clearly ischemic
Signs of cerebral ischemia (stroke, hemiparesis, altered consciousness)	Wound proximity to major vessels
Severe hypotension or shock not explained by other injuries	Minor hemoptysis or dysphagia
Massive hemoptysis or hematemesis	

In our case, the patient was hemodynamically stable, so a CT angiogram was performed, after which the patient was shifted to the OR for definitive management.

Imaging studies

While physical examination is vital, imaging modalities such as CT angiography are considered the gold standard for comprehensive assessment of vascular integrity and the extent of injury [2].

Historically, all penetrating neck injuries with platysma penetration underwent mandatory exploration. Modern trauma practice favors selective management guided by imaging and clinical findings.

Management protocols

Following initial resuscitation, damage control surgery may be necessary to address vascular injuries, particularly if active bleeding is present. The incision is guided by an imaginary line between the earlobe crease and the sternal notch, although identifying anatomical landmarks can be challenging due to hematoma expansion. Once a carotid artery injury is identified, proximal and distal control must be established rapidly. Median sternotomy may be necessary for common carotid artery injuries, while external carotid artery injuries may require dissection at the bifurcation. Repair strategies include minimal debridement and direct suturing for small injuries, which was done in our case, while larger segmental injuries may necessitate interposition grafts using saphenous vein or PTFE. Ligation is considered in cases of worse patient condition, associated severe brain injuries, or extensive distal thrombosis, with a focus on life-saving measures over complex reconstructions [7].

Outcomes generally favor surgical repair over ligation, with significant rates of neurological improvement noted [8].

After surgical repair of a penetrating carotid artery injury, the use of antiplatelet therapy is selective and depends on the type of repair and bleeding risk. In patients who undergo primary repair, patch angioplasty, or interposition grafting and achieve secure hemostasis, antiplatelet therapy, most commonly aspirin, is often initiated postoperatively to reduce the risk of thrombosis and embolic stroke, particularly when there has been intimal injury or endothelial disruption. Antiplatelets are routinely indicated after carotid patch angioplasty or prosthetic graft placement once the risk of surgical bleeding is controlled. In contrast, antiplatelet therapy is not routinely required after simple vessel ligation and is delayed or avoided in patients with ongoing bleeding, coagulopathy, or associated injuries with high hemorrhagic risk. Overall, postoperative antiplatelet therapy is considered when the benefit of preventing carotid thrombosis and stroke outweighs the risk of bleeding, and it is generally started after hemostasis is confirmed and the patient is hemodynamically stable [1,4].

Accordingly, our patient was considered low risk for bleeding and was discharged on dual antiplatelet therapy to reduce the risk of thrombosis and its consequences.

Complications

Complications arising from carotid artery injuries include ischemic stroke due to embolism or hypoperfusion, hemorrhage either from the initial injury or subsequent surgical intervention, and neurological deficits resulting from direct nerve injury or ischemia [9].

Prognosis

The prognosis for patients with neck gunshot wounds and carotid artery injuries varies significantly based on injury severity, timeliness of intervention, and the presence of concomitant injuries. Early recognition and management are critical to improving outcomes [9].

## Conclusions

In cases of neck gunshot wounds with potential carotid artery involvement, a structured approach combining thorough assessment, adherence to ATLS protocols, and timely surgical intervention is essential to minimize morbidity and mortality.
